# Effect of Dairy, Season, and Sampling Position on Physical Properties of Trentingrana Cheese: Application of an LMM-ASCA Model

**DOI:** 10.3390/foods11010127

**Published:** 2022-01-05

**Authors:** Michele Ricci, Flavia Gasperi, Isabella Endrizzi, Leonardo Menghi, Danny Cliceri, Pietro Franceschi, Eugenio Aprea

**Affiliations:** 1 Center Agriculture Food Environment, University of Trento, Via E. Mach, 1, 38010 San Michele all’Adige, Italy; michele.ricci@unitn.it (M.R.); flavia.gasperi@unitn.it (F.G.); leonardo.menghi@unitn.it (L.M.); danny.cliceri@unitn.it (D.C.); 2 Research and Innovation Centre, Fondazione Edmund Mach, Via E. Mach, 1, 38010 San Michele all’Adige, Italy; isabella.endrizzi@fmach.it (I.E.); pietro.franceschi@fmach.it (P.F.); 3 Department of Technology and Innovation, University of Southern Denmark, Campusvej 55, 5230 Odense, Denmark

**Keywords:** hard cheese, dairy technology, quality control, texture analysis, linear mixed models, multivariate analysis

## Abstract

Trentingrana hard cheese is a geographic specification of the PDO Grana Padano. It is produced according to an internal regulation by many cooperative dairy factories in the Trentino region (northern Italy), using a semi-artisanal process (the only allowed ingredients are milk, salt, and rennet). Within the PSR project TRENTINGRANA, colorimetric and textural measurements have been collected from 317 cheese wheels, which were sampled bi-monthly from all the consortium dairies (*n* = 15) within the timeframe of two years, to estimate the effect on physical properties related to the season of the year and the dairy factory implant. To estimate the effect of the dairy and the time of the year, considering the internal variability of each cheese wheel, a linear mixed-effect model combined with a simultaneous component analysis (LMM-ASCA) is proposed. Results show that all the factors have a significant effect on the colorimetric and textural properties of the cheese. There are five clusters of dairies producing cheese with similar properties, three different couples of months of the year when the cheese produced is significantly different from all the others, and the effect of the geometry of the cheese wheel is reported as well.

## 1. Introduction

Trentingrana cheese is an extra-hard-seasoned cheese type with Italian Protected Designation of Origin (PDO) falling under the Grana Padano PDO [1]. The Trentingrana trademark, embossed on the wheel near the “Grana Padano” label, emphasizes the distinctive properties of this cheese [2]; this includes the use of raw cow milk only from livestock on mountain terrains exclusively in a delimited area and the application of restricted cattle feeding and cheese manufacturing protocols [3].

Texture properties of extra-hard cheese affect how the cheese is portioned and packed. Texture also affects the behavior of the cheese when it is subjected to shredding or grating, and how the cheese retains gas and hence, its predisposition to form eyes, cracks, or swell. The color of cheese significantly contributes to sensory responses and plays an important role in the anticipation phase of selection and consumption of food materials [4]. Consumer expectations are influenced both by cheese color itself and its homogeneity. Both colorimetric and textural properties are critical for the commercial value of hard seasoned cheese, such as Trentingrana cheese. The measurement of those properties defines the product from a technological and commercial point of view.

The quality of the raw milk (casein content, casein micelle structure, and integrity) [5,6] and conditions of the cheese-making process (for example, pre-acidification of milk, type and quantity of rennet, cooking temperature, acidification of the cheese mass, and temperature and humidity during seasoning) [7] are fundamental factors that influence the textural and colorimetric properties of cheese [8].

The production chain of hard seasoned cheese with Protected Designation of Origin (PDO) usually exists in many individual dairy factories belonging to the same producer cooperative that transforms raw milk conferred daily from many small farms. This fragmentation of the process suggests that there may be significant differences in the process and the characteristics of the raw material [9] despite the presence of a consortium that regulates the production. At a supply chain level, the effect of process and raw materials conditions on the physical characteristics of the final product are mostly attributable to the dairy factory and the time of the year when the fresh milk is delivered.

Due to the large dimension of the Trentingrana cheese wheel (a height from 20 to 26 cm and a diameter from 35 to 45 cm), these physical properties can vary according to the position in the cheese wheel, since water content and temperature in the early stage of the process, as well as microbial activity, depends on the distance from the center. Thus, for a more comprehensive description of the physical properties of a single cheese wheel, it is necessary to take multiple samples from the same cheese wheel, addressing the distance from the central position.

To date, there are few studies that have investigated the effect of the dairy factory and the time of the year on the colorimetric and textural properties of hard cheeses; nevertheless, Bellesia et al. [10] highlight a large variability among dairies for volatile components of Parmigiano Reggiano cheese while Careri et al. [11] report a much lower variability on the same type of cheese in relation to chemical parameters and non-volatile fractions. Franceschi et al. [12] notice how the month of the year and the dairy can determine the efficiency of the cheese-making process. According to our knowledge, no study has tried to estimate the effect of different dairies and different months of the year on the textural and colorimetric properties of cheese.

To estimate the effect of the dairy factory and the time of the year on both colorimetric and textural properties while taking into account the natural variability of artisanal cheese production, it is necessary to characterize a real-scale production process. From a statistical point of view, the optimal data analysis strategy has to be able: (a) to take into account the multilevel nature of the experimental design separating the contribution of the different study factors from the variability arising during the cheese wheel making process; (b) to estimate the correlation among colorimetric and textural properties.

Linear mixed models (LMM) are an extension of standard linear models for regression analysis of experimental designs containing observations that cannot be assumed to be independent of each other, such as repeated measurements or measurements from the same sample [13]. This method takes into account the error structure in the data, estimating at the same time the effect of fixed factors, which are the factors of interest, and random factors, which represent the individual variability caused by non-measurable sources of variation. In our specific scenario, linear mixed models can handle information better than an ANOVA, because they take into consideration repeated measurements in the same cheese wheel and they describe in the models the variability related to uncontrolled production parameters.

LMM is a univariate approach that can be integrated into a multivariate framework extending the principle of ANOVA simultaneous component analysis (ASCA) [14], which consists of applying a multivariate matrix decomposition (based on SVD) on the matrices of the expected values estimated from a univariate analysis of the experimental variables. In other words, the ASCA approach analyzes at a multivariate level the effect of each factor, and uses the powerful visualization instruments of PCA, such as score and loading plots, to highlight the latent factors that are contributing to each design factor.

The application of linear mixed models with ASCA decomposition in an experimental design with nested and random factors and unbalanced levels is still an uncommon procedure in data analysis. Therefore, the issues related to the evaluation of this type of statistical model and its application are still not widely addressed in the context of food manufacturing quality control. In the last decade, the interest in this methodology has been increasing, due to the growing availability of vast multivariate datasets from stratified experimental design acquired in ecological and industrial studies.

Stamirova et al. [15] applied a linear mixed model to evaluate differences in food manufacturing, presenting a statistically robust procedure for analyzing data on the effect of different herbal tea pasteurization treatments from three different years of production. The effect of many confounding factors in the chemical properties of a herbal product is estimated using a statistical model to describe the complex interactions between the main fixed and random factors, obtaining consistent results.

Martin and Govaerts [16] reviewed several different applications of linear mixed models with ASCA decomposition for different datasets, ranging from metabolomics to sensory science. A step-by-step procedure to develop and evaluate a statistical model with both random and fixed effects is explained, and the procedures to estimate models’ outcomes, such as statistical significance and effect size are compared. A suitable procedure for matrix decomposition and reconstruction is presented, with a focus on the individuation of the most important effects after the transformation of data.

This study aims to analyze the variation of textural and colorimetric parameters of a semi-artisanal PDO product according to dairy, sampling position, and time of the year using LMM-ASCA analysis, which has proven to be a valid statistical procedure to evaluate a large-scale dataset of measurements from Trentingrana industrial quality control process.

## 2. Materials and Methods

### 2.1. Sampling Procedure

During the years 2017 and 2018, a total of 317 Trentingrana cheese wheels were collected from the 15 dairies belonging to Trentingrana Consortium.

According to the procedure described in Endrizzi et al. [2], every 2 months, 1 to 3 cheese wheels were randomly sampled from the 18-month-ripened “first-quality” wheels produced by each dairy during the considered sampling period of 2 months. 

The number of cheese wheels sampled from each dairy factory was determined according to its volume of production during the two months: one wheel for each dairy delivering up to 1000 wheels, two wheels for 1001 to 1500 delivered, and three for more than 1500 delivered cheese wheels.

Due to the internal organization procedures of the Trentingrana consortium, the cheese wheels sampled during the last couple of months had a ripening period of three weeks less than the others.

All the measurements were acquired on a weekly basis: a subset of 6 cheese wheels was brought from the storehouse to the laboratory of the Edmund Mach Foundation, where each wheel was opened and visually evaluated by a panel of experts. Then, each cheese wheel was portioned. One portion was evaluated by the quality control panel of the Trentingrana consortium, the other one was directed to instrumental analysis.

**Figure 1:**
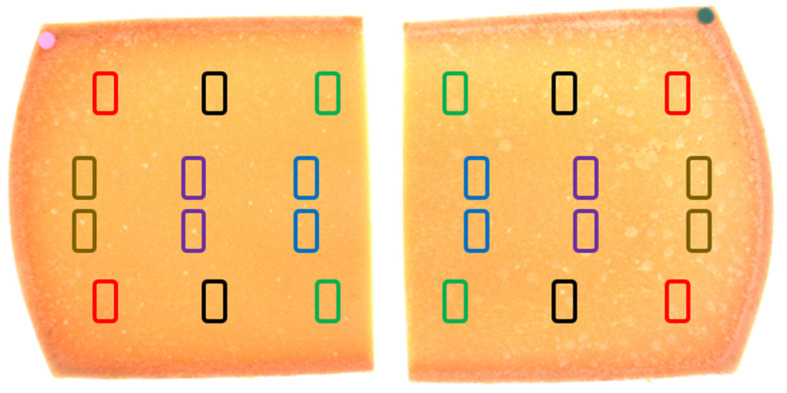
Example of two slices of Trentingrana cheese. Each box corresponds to one sampling position, each color to a sampling zone: round (RND, red); round central (RNDs, brown); external plate (RNDp1, black); intermediate zone (Int, purple); internal plate (RNDp2, green); center (CNT, blue).

Subsequently, from each wheel, 24 blocks of cheese were sampled, each block with a length of 3 cm, a width of 1.5 cm, and a height of 1.5 cm. Each set of blocks from the same product was cut at the same distance from the center of the wheel and assigned to one of six different categories according to the distance from the central position. The sampling position is illustrated in Figure 1. Colorimetric and textural analyses were carried out on each block.

Globally, 317 cheese wheels were sampled from 12 sampling sessions from each dairy on a bi-monthly basis and 7608 measurements were acquired for 6 different parameters during 54 analytical sessions.

### 2.2. Analytic Determinations

#### 2.2.1. Color

L*a*b components from the CIELAB color space model [17] were measured once on one of the wider surfaces of each cheese block sample using a CR-400 colorimeter (Konica Minolta Sensing Inc., Tokyo, Japan) using the D65 illuminant source, an observation angle of 2°, and previously calibrated with a reference white standard ceramic tile. Data were acquired using the CM-S100w SpectraMagicTM color data software (Konica Minolta Sensing Inc., Tokyo, Japan).

#### 2.2.2. Textural Properties

Texture properties were measured on each cheese block by a TA-XT texture analyzer (Stable MicroSystem Ltd., Godalming, UK) applying a uniaxial compression/penetration on one of the wider sides of the cheese block sample. Following the method described by Noël et al. [18], a 4 mm probe was used with a speed of 1.67 mm/s, a trigger force of 5 N, setting the endpoint of the measurement when a maximum strain of 90% of the height of the sample was obtained, and three mechanical parameters were calculated on the recorded curves. Those parameters are shown in Table 1.

### 2.3. Statistical Analysis

Due to the various sources of variability that affect the final product, the results are highly-structured data. The procedures to analyze those data require the selection of a suitable model for the design of the experiment, a reliable procedure to evaluate the significance of each factor analyzed, and a dimensional reduction technique to summarize all the information [14]. Before each analysis, each variable has been centered and scaled to unit variance, to obtain comparable results from each model. Each variable was checked for the assumption of normality using QQ plots (Appendix A).

### 2.4. Model Selection

The experimental design was built to take into account the many specific sources of variation inside the supply chain of a PDO product [2]. Here we wanted to estimate the effect of the dairy factory and the time of the year (“Time” factor, from now). The position in the wheel was also included in the model to estimate the natural variability present in each cheese wheel.

To describe the design of the sampling campaign, we used an unbalanced experimental design with four factors and their interactions, represented by this Equation (1):

(1) $$x_{i_{klnt}}=\mu_{}+\alpha_{k}+\beta_{l}+\gamma_{g}+{\left(\alpha \beta \right)}_{kl}+{\left(\alpha \gamma \right)}_{kg}+{\left(\beta \gamma \right)}_{lg}+\delta_{t}+\epsilon_{klgti}$$

where *α* corresponds to the fixed factor “sampling position” with level *k*, *β* corresponds to the fixed factor “Time” with level *l*, *γ* to the fixed factor “dairy factory” with level *n*, and *δ* to the random factor “cheese wheel” at level *t*. Level *i* represents the repeated measurements conducted in the same sampling position of the same cheese wheel from different blocks. This model is applied to each colorimetric and textural parameter estimated.

Because each couple of months the cheese wheel has been analyzed from each dairy, the cheese wheel factor is nested inside the other fixed factors, so there is no interaction effect that involves this factor.

### 2.5. Permutation Test for Linear Mixed Models’ Significance

For each variable, a linear mixed model was estimated in order to assess the significance of the factor “Time”, “dairy factory”, and “position”. The cheese wheel factor was defined as random. For each factor and each interaction, the matrix of contrasts was set to obtain a sum to zero estimation. This procedure ensures that each level would be tested with the grand mean of the dataset.

As previously stated, the aim of the experiment was to determine the effect of the dairy factory (*n* = 15) and the time when cheese is produced (*n* = 6). For each factor and interaction, the matrix of contrasts was set to obtain a sum to zero estimation, to ensure that each level is compared with the grand mean.

Each model was validated by a permutation test. The null hypothesis for each variable was that there is no significant effect for any of the parameters except for the random factor of the cheese wheel. Montecarlo simulation (*n* = 1000) was used to estimate the null hypothesis’ distribution of the sum of squares decomposition and to compare the results to the effective decomposition, as discussed in Stamirova et al. [15]. Each permutation test was performed, maintaining the nested structure of the factor of the sampling position inside the other two factors, to make sure that the null hypothesis’ distribution estimated was attributable only to the effect of the factors investigated and their interactions. The α-values chosen are 1/1000 and the null hypothesis is rejected when the permuted sum of squares decompositions is lower than the values obtained by the real model for less than 5% of the permutations. After the permutation, for each factor, the estimated *p* values were adjusted using the Bonferroni correction.

Using multiple permutations at the univariate level, after an appropriate adjustment for multiple comparisons, instead of a singular permutation test at the multivariate level allows for avoiding the effect of internal correlation when evaluating the significance of each parameter.

### 2.6. Estimation of the Model

All the significant effects for each level of each factor are estimated using the Equation (2):

(2) $$X_{aj}=T_{aj}*P_{aj}$$

where $X_{aj}$ is the effect matrix of the factor *a* in the *j*-th variable, *T* is the contrast matrix, and *P* is the vector of the effects. The effect matrix is grouped for all the parameters to obtain a complete effect matrix that represents the decomposition of the effect estimated for each level of a factor for every variable. To observe the distribution of the samples and to interpret their relations with each variable, PCA analysis is adopted on the matrix of effects.

Before the estimation of principal components, to compare the effects estimated for different variables, the matrices of effects of each factor were centered for each variable.

This sub-model summarizes the results of all the univariate models in a small number of principal components and highlights similarities and differences between levels using the biplot graphical tool to represent projection on principal components of both variables and levels.

### 2.7. Statistical Software

All the statistical analyses have been performed using R version 4.1.0 [19], the lme4 package version 1.1-27.1 [13], the ggpubr package version 0.4.0 [20], and the factoextra package version 1.0.7 [21].

## 3. Results and Discussion

### 3.1. Linear Mixed Models

According to the results of the permutation test, the factors “Dairy Factory” and “Sampling Position” are significant in the models estimated for all parameters, and the factor “Time” is significant for each parameter except for colorimetric index a* and the textural parameter “Area under the curve”. The interactions between “Dairy Factory” and “Time” are not significant for all the parameters, the “Time” and “Sampling Position” interaction is significant only for the colorimetric parameters, and the interaction between “Dairy Factory” and “Sampling Position” is significant for each parameter except the elastic modulus.

Those results state that the color and the structure of the cheese wheels change when there are changes in the production process, in the sampling position, and, to a lower amount, the season of the year when milk was produced.

**Figure 2:**
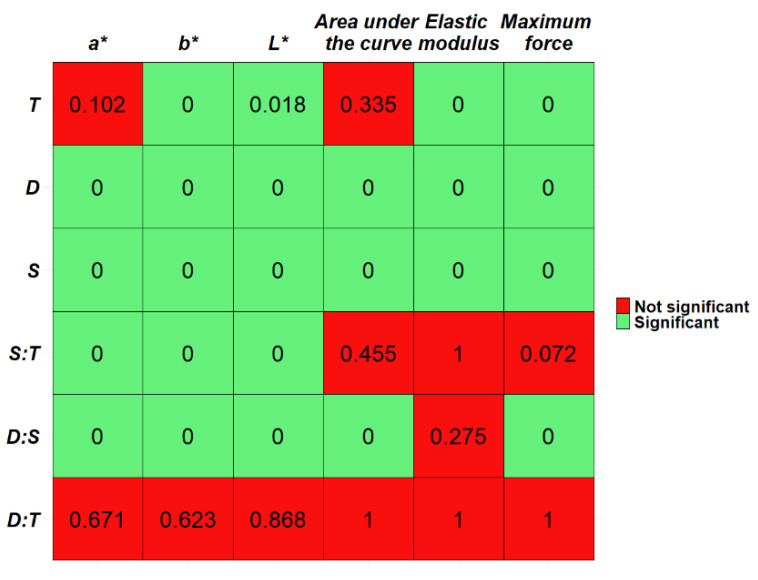
Tile plot reporting the p values estimated from the permutation tests for the significance of each factor (“T”: Time, “D”: Dairy Factory, “S”: Sampling Position, “S:T”: interaction between Sampling Position and Time, “D:S”: interaction between Dairy Factory and Sampling Position, “D:T”: interaction between Dairy Factory and Time) of the linear mixed models.

There are no important differences between the variations of each dairy at different seasons, but the overall value of a sampling position varies according to the dairy and the time, at least for colorimetric properties. Results are shown in Figure 2.

### 3.2. Simultaneous Component Analysis: LMM-ASCA Results

**Figure 3:**
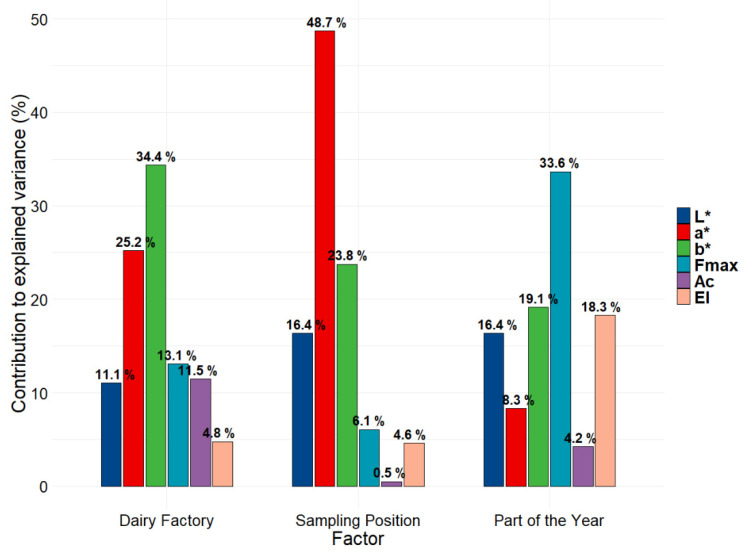
Barplots showing the contribution of each parameter to the explained variance for each LMM-ASCA decomposition of the model.

**Figure 4:**
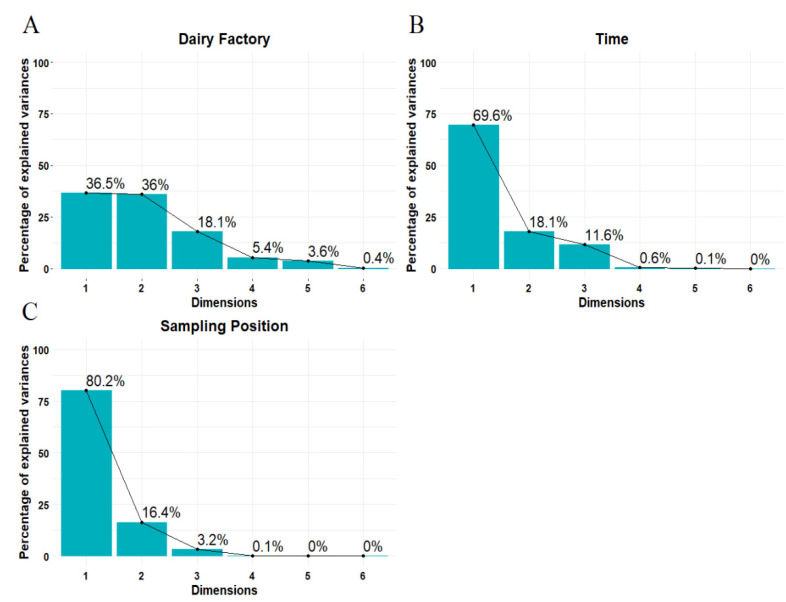
Scree plots for the ASCA decomposition of the factors “Dairy Factory” (**A**), “Time” (**B**), and “Sampling Position” (**C**).

The first features of the model evaluated were the estimations of the contribution of each parameter to the explained variance for each different factor, as reported in Figure 3. The contribution of each parameter to the multivariate decomposition of each factor was estimated according to Kassambara [22]. The contribution of explained variance was estimated for all the dimensions considered for each factor: four for “Dairy Factory” and two for “Sampling Position” and “Time”. The number of dimensions taken into consideration was estimated to obtain at least 85% of explained variance for each factor; the values are represented in the scree plots in Figure 4.

In the LMM-ASCA model, the contributions of the experimental variables represent the effective fraction of the variance considered for each factor due to the preprocessing step of unit scale normalization of each parameter before the estimation of all the models, and the estimation of the effect matrix from all models together.

Different from PCA, principal components from ASCA decompositions represent the effective percentage of explained variance quantitatively, and the contribution of each parameter is quantitative information of which the parameter effectively varies the most between the levels of each factor considering the effect of all the other factors in the experimental design.

The parameters containing the largest amount of variance in ASCA decomposition for the factor “Dairy Factory” are the colorimetric parameters and, for a less amount, the textural parameter Area Under the Curve (Ac); for the factor “Sampling Position”, the colorimetric indices contribute more, and for the factor “Time”, the textural parameters are the most important in determining the overall variance together colorimetric index b*.

#### 3.2.1. Dairy Factory

Results of the ASCA multivariate analysis show that 96% of explained variance is described by the first four principal components, as shown in the scree plots in Figure 4.

The first component describes 36.5% of the total variation. This component describes mostly the variation from the overall mean for the colorimetric component a*, then the variation for the component L*, and then the anticorrelated variation to those colorimetric properties of all the textural parameters.

**Figure 5:**
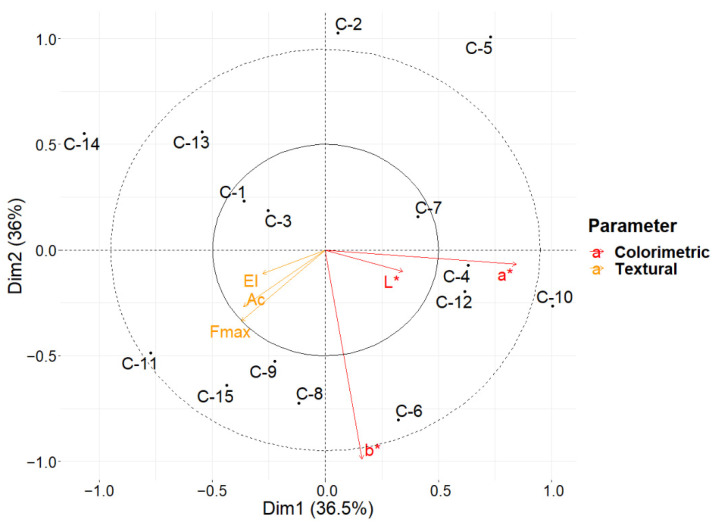
Biplot for ASCA showing the values for the first two principal components for the LMM-ASCA decomposition to the factor “Dairy Factory”. Score values for each level are represented by black dots and labeled from C-1 to C-15 for each dairy factory. Loading values are represented using arrows for each parameter acquired, and colored according to the type of the measurement: “El” stands for elastic modulus; “Ac” for the area under the curve; “Fmax” for maximum force; “L*”, “a*”, and “b*” labels are referred to the coordinates of the lab color spaces.

According to this component, as reported in the biplot in Figure 5, the dairies can be ideally divided for the overall value of all the textural parameters and the value of the colorimetric parameters L* and a* of the cheese wheels they produced. In the leftmost part of the graph, there are the dairies labeled C-14 and C-11, which produced cheese are characterized by overall higher values for all the textural properties and less bright color of the grain; in the central part, between the score values of −0.55 and 0.55 of the first principal component, there are the dairies C-1, C-2, C-3, C-6, C-7, C-8, C-9, C-13, and C-15, which produced cheese with textural parameters and L* and a* colorimetric parameters not significantly different from the overall mean; in the rightmost part of the graph there are the remaining dairies (C-4, C-5, C-10, C-12), which produced cheese wheels having textural parameters lower than the overall mean and higher values of L* and a* colorimetric values than the overall mean.

The second component, which accounts for 36% of the total variance, represents mostly the variation related to the colorimetric index b*. In the higher part of the graph, there are the dairies C-2, C-5, C-13, and C-14, those having cheese wheels with lower values of the colorimetric parameter b* than the overall mean; between the score values of −0.55 and 0.55 are the dairies C-1, C-3, C-4, C-7, C-9, C-10, C-11, and C-12, which produced cheese wheels having an average value of b* not significantly different from the overall mean; in the lower part of the graph are the dairies labeled C-6, C-8, and C-15, which produced cheese wheels having an average value of b* higher than the overall mean. 

The first two principal components show that there are four dairy factories that differ from the overall mean by more than 95% of the explained variance, which are C-2, C-5, C-10, and C-14.

**Figure 6:**
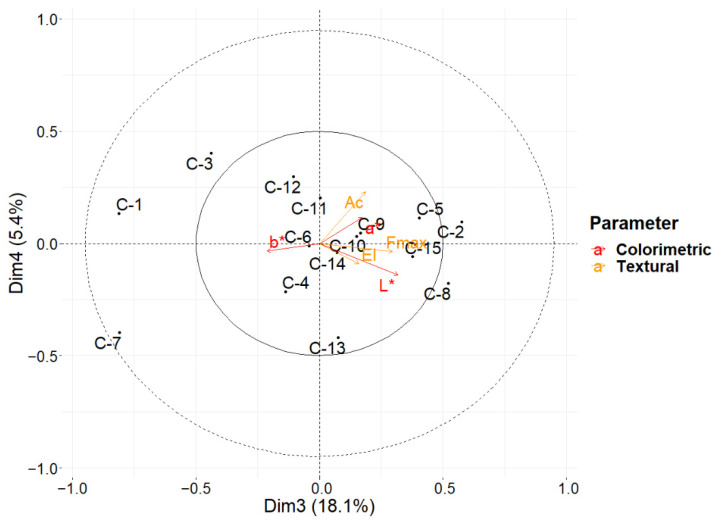
Biplot for ASCA showing the values for the third and fourth principal components for the LMM-ASCA decomposition to the factor “Dairy Factory”. Score values for each level are represented by black dots and labeled from C-1 to C-15 for each dairy factory. Loading values are represented using arrows for each parameter acquired, and colored according to the type of the measurement: “El” stands for elastic modulus; “Ac” for the area under the curve; “Fmax” for maximum force; “L*”, “a*”, and “b*” labels are referred to the coordinates of the lab color spaces.

The third principal component describes the 18.1% of the overall variance (Figure 6) and is determined mostly by the values of all the physical parameters, with the colorimetric b* index in anticorrelated position with respect to all the other parameters. This component divides three dairies (C-1, C-3, C-7) from all the others, for their overall lower values of textural and colorimetric parameters, except b*.

The fourth principal component explains only 5.4% of the total variance, and it describes the variation mostly related to the textural index of the area below the curve and, in a lower amount, to the a* colorimetric index. It divides mostly the dairies C-2 and C-5 from the overall mean. The latter two principal components explain 23.5% of the overall variance and highlight another interesting cluster including four dairies C-6, C-9, C-10, and C-14.

**Figure 7:**
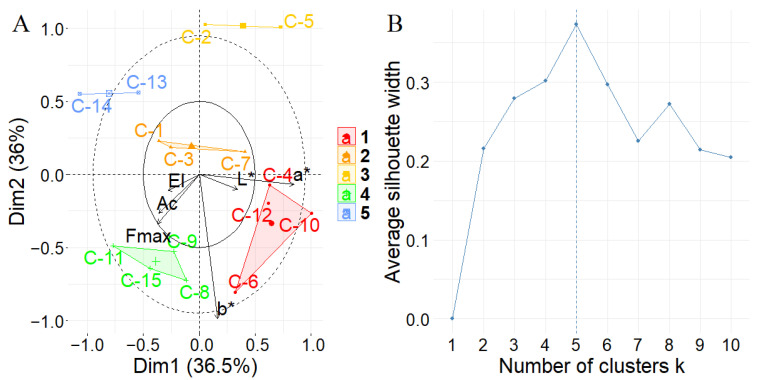
Results of the clustering analysis on LMM-ASCA decomposition of the “Dairy Factory” factor; (**A**) representation on clusters; (**B**) results of the Elbow method for the estimation of the optimal number of clusters. Score values for each level are represented by black dots, and labeled from C-1 to C-15 for each dairy factory, and colored according to the cluster estimated. Loading values are represented using arrows for each parameter acquired: “El” stands for elastic modulus; “Ac” for area under the curve; “Fmax” for maximum force; “L*”, “a*”, and “b*” labels are referred to the coordinates of the lab color spaces.

To estimate the presence of cluster inside the ASCA decomposition for this factor, which, differently than the other two analyzed, has many different levels and more structured results, a k-means cluster analysis has been performed on the score value matrix of the LMM-ASCA decomposition. The clustering procedure was performed using the Hartigan-Wong algorithm reported by Hartigan [23]; the optimal number of clusters was estimated using the Silhouette method as reported by Kassambara [24]. Results are summarized in Figure 7.

Cluster analysis highlights the presence of two big clusters, named 1 and 5, both containing four dairies. Then it is estimated that cluster 2, which contains three different dairies with values near to the center of the plot (C-1, C-3, and C-7), and two small clusters, that represents the presence of the dairy factories have values very different from the overall mean (C-2 and C-5 in cluster 3; C-13 and C-14 in cluster 4).

Differences in the properties of the final product can be explained by differences in the raw material and the technological process: together with the quality of raw milk used, the type and quantity of rennet and natural whey starters, the handling of the curd, the operations of acidification, heating, cooling, and the ripening condition and time all these factors are able to affect the quality of the final product. Even though the production of Trentingrana cheese follows a general standardized procedure and the Trentingrana cheese disciplinary reports the crucial technological steps, it is a semi-artisanal production, with a significant internal variability related to the different dairy.

The clusters estimated can be partially explained because of important similarities in the technology of the process: The dairy C-1 and C-3 use both the same kind and the same quantity of rennet, and they have similar values of time and temperature in the procedure of the heating of the curd (data not shown).

Differences between dairy factories belonging to the same consortium have already been detected by Franceschi et al. [12] for the efficiency of the process, and Mucchetti et al. [7] noticed the effect of slight variation inside the production process of extra-hard cheese as an important source of variation for the appearance and the structure of the cheese; our research also highlighted differences in the physical properties of the cheese wheels that can be used as quality indices.

#### 3.2.2. Time

**Figure 8:**
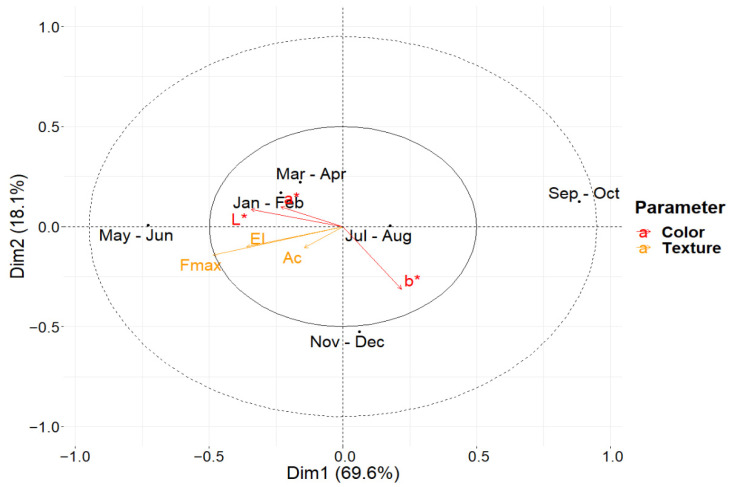
Biplot for ASCA showing the values for the first two principal components for the LMM-ASCA decomposition to the factor “Time”. Score values for each level are represented by black dots, labeled using the first three letters of the two months of each couple of months when the cheese wheels were sampled. Loading values are represented using arrows for each parameter acquired, and colored according to the type of the measurement: “El” stands for elastic modulus; “Ac” for area under the curve; “Fmax” for maximum force; “L*”, “a*”, and “b*” labels are referred to the coordinates of the lab color spaces.

For the factor “Time”, ASCA decomposition represents 87.1% of total variance with the first two principal components. As shown in the biplot in Figure 8, the first PC describes variations in all the colorimetric parameters, with b* in an anticorrelated position with a* and b*, and, with less significance, the textural parameters elastic modulus and maximum force applied. The second principal component describes variation mostly due to the colorimetric index L* and the elastic modulus. Along with the first component, the wheels sampled in May and June are characterized by brighter and thicker grain than the average, the wheels sampled from January to April, in November and December, and in July and August are characterized by medium values of textural and colorimetric properties, and the wheels sampled from September to October show higher values of the textural parameters and more yellow color than the average. The second component separates the wheels produced from November to December from all the others because of a significantly higher value of the colorimetric index b* that can be interpreted as a more yellowish color than the average. 

Differences and similarities found in this analysis are likely to highlight the effect of seasonal variation in the raw milk used, which causes a change in the overall content of protein (data not shown). The overall lower values of density of the last two couples of months (November and December) may partly be due to the sampling plan. Indeed, the cheese wheels produced over that period were seasoned 3 weeks less than the others to ease the logistic organization of the sampling procedure within the consortium.

#### 3.2.3. Sampling Position

**Figure 9:**
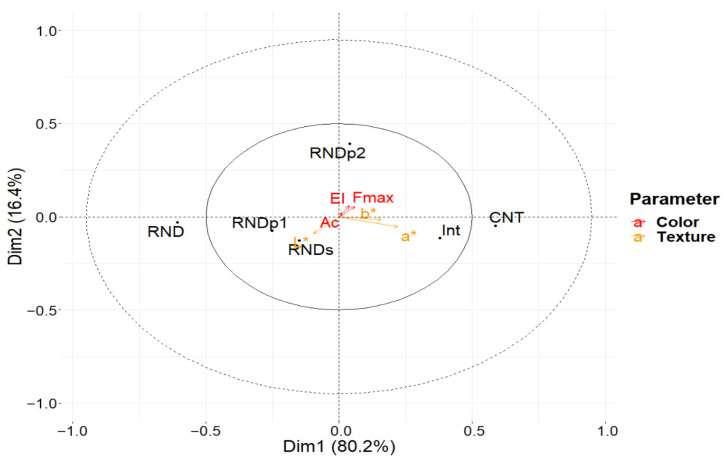
Biplot for ASCA showing the values for the first two principal components for the LMM-ASCA decomposition of the factor “Sampling Position”. Score values for each level are represented by black dots, each label corresponds to a different sampling position: “RND”: round; “RNDs”: round central; “RNDp1”: external plate; “Int”: intermediate zone; “RNDp2”: internal plate; “CNT”: center. Loading values are represented using arrows for each parameter acquired, and colored according to the type of the measurement: “El” stands for elastic modulus; “Ac” for area under the curve; “Fmax” for maximum force; “L*”, “a*”, and “b*” labels are referred to the coordinates of the lab color spaces.

Multivariate ASCA estimated 96.4% of the explained variance with two principal components. The first component, as reported in the biplot in Figure 9, describes the overall variation related to all textural and colorimetric parameters, with a strong anticorrelation between the colorimetric parameter L* and all the other measurements.

In this component, the sampling position levels are placed from left to right according to higher values of texture and a darker color. This disposition corresponds to a decreasing distance from the center of the cheese wheel.

The second component describes mostly variation in the colorimetric indices and the area under the curve. According to this component, all the levels but one do not have a significant variation from the grand mean, except for the intermediate position between the central zone and the round of the wheel, which results in higher values for each index. This disposition in the biplot suggests that the gradient highlighted by the first component is not linear and that the intermediate position has higher values than those expected according to the variation of the distance from the center.

Differences due to the sampling position inside the cheese wheels are well known [25], they are due to the inhomogeneous content of water, protein, and fat in the casein structure, related to the diffusion phenomenon during curing and seasoning of the cheese wheel. Water, which spreads from the center to the external zone of the wheel when the evaporation phenomenon begins, acts as a low-viscosity lubricant, reducing overall texture properties and as a solvent for coloring molecules, reducing their concentration, hence the color brightness decrease [26]. The color difference is mostly related to the concentration of products of the Maillard reaction naturally occurring in cheese [27]: the central zone of the cheese wheel maintains a temperature above 50 °C longer than the external zones, therefore more chromophore molecules are formed during and after the heating of the curd [28].

## 4. Conclusions

Significant differences have been detected among the dairy factories of the Trentingrana consortium, the time of the year, and the sampling positions. Analysis of significant differences in the context of real-scale quality control at an aggregate level allows for detecting which are the phenomena that may modify the physical properties of cheese, to allow improvements in the production process, and most importantly, to focus on what are the features that affect the production process and to understand them better.

Those results are related to the effect of variations of the process of production on the physical properties of the final product and can be detected when changes in the three factors considered (dairy factory, time, and sampling position) have an overall significant effect. The differences in the percentage of explained variance by each different factor evidenced the importance of the sampling position as a critical point in the evaluation of cheese wheels: the results highlighted a large effect due to sampling position, especially on color values. Research on these products should consider the importance of the geometry of the cheese wheel to characterize its overall properties.

Differences detected for the dairy factory factor are more difficult to interpret. Results show that there are similarities and differences related to the production process, as resulted from information given by the consortium. At the same time, raw material properties are not always easily related to the final product, and this may also be explained by the importance of the technologies applied for the caseification process, as, for example, the use of curd in liquid or in powder form, or a mixed combination, its coagulation activity, and the gradient of temperature during the heating of the paste.

Differences related to the time when the cheese wheels were produced are likely related to the seasonal variation of the raw milk, which tends to have different protein content and a different concentration of beta-carotene depending on the season and to a different diet, causing variations in the textural and colorimetric properties.

Quality evaluation of hard seasoned cheese requires many different measurements to define all its properties, from physical to sensory. In this study, LMM-ASCA procedures allow for analyzing the effect of multiple different factors comparing many measurements at the same time, giving a statistically valid comparison of the whole profile of the product between different subgroups in complex or nested experimental designs.

The results of those analyses are functional to the development of classification models for the quality monitoring of the production of hard seasoned cheese and to evaluate the effect of the process of production on the commercial quality of the product Trentingrana Cheese.

At this moment, further research is needed to better understand the relationship between the variation in the production process and the physical quality of hard seasoned cheese.

## Figures and Tables

**Table 1:** Parameters extrapolated from the stress/strain curve estimated from uniaxial compression.

Parameter	Description	Measure Unit
Maximum Force (Fmax)	The maximum amount of force applied by the uniaxial probe to the sample.	N
Area under the curve (Ac)	The whole area under the force/strain curve during the compression of the sample until the endpoint.	N*mm
Elastic modulus (El)	The slope of the linear part of the stress-strain curve.	N/mm

**Figure A1:**
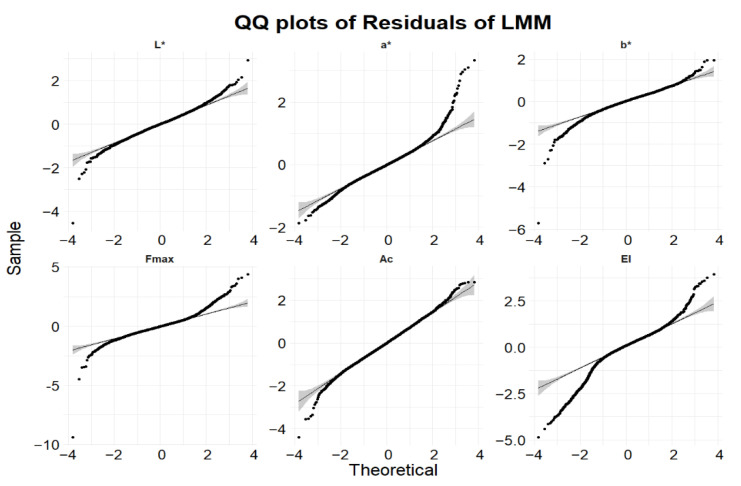
Q-Q plot of the normal distribution of each parameter.

## Data Availability

The datasets supporting the current study are confidential and, thus, not publicly available. Nevertheless, according to the policies of the Trentingrana consortium, data could be available from the corresponding author upon reasonable request.
